# Effect of Presence versus Absence of Hypertension on Admission Heart Rate-Associated Cardiovascular Risk in Patients with Acute Coronary Syndrome

**DOI:** 10.1155/2022/3001737

**Published:** 2022-02-14

**Authors:** Yihua Xia, Zhijian Wang, Fei Gao, Lixia Yang, Jing Liang, Dongmei Shi, Yujie Zhou, Xiaoteng Ma

**Affiliations:** Beijing Anzhen Hospital, Capital Medical University, Beijing Institute of Heart Lung and Blood Vessel Disease, The Key Laboratory of Remodeling-Related Cardiovascular Disease, Ministry of Education, Beijing, China

## Abstract

**Background and Aims:**

Heart rate (HR) and hypertension are both important risk factors for adverse cardiovascular (CV) events in patients with established coronary artery disease (CAD). We sought to evaluate whether hypertension can modify the effect of admission HR on adverse CV events in patients with acute coronary syndrome (ACS).

**Methods:**

A total of 1056 patients with ACS undergoing percutaneous coronary intervention (PCI) were analyzed. All patients were classified into three groups according to the tertiles of admission HR (T1: ≤66 bpm, *n* = 369; T2: 67–73 bpm, *n* = 322; and T3: ≥74 bpm, *n* = 365). The primary endpoint was defined as major adverse CV events (MACEs), including all-cause death, stroke, myocardial infarction, or unplanned repeat revascularization. The multivariate Cox regression model was performed to evaluate the association of admission HR with MACE stratified by hypertension.

**Results:**

During the median follow-up of 30 months, a total of 232 patients developed at least one event. After adjusting for other covariates, elevated admission HR was significantly associated with an increased risk of MACE only in patients with hypertension (when T1 was taken as a reference, the adjusted HR of T2 was 1.143 [95% CI: 0.700–1.864] and that of T3 was 2.062 [95% CI: 1.300–3.270]); however, in patients without hypertension, admission HR was not associated with the risk of MACE (when T1 was taken as a reference, the adjusted HR of T2 was 0.744 [0.406–1.364] and that of T3 was 0.614 [0.342–1.101]) (*P*=0.025 for interaction).

**Conclusions:**

In patients with ACS undergoing PCI, the association of elevated admission HR with an increased risk of MACE was present in individuals with hypertension but not in those without hypertension. This finding suggests a potential benefit of HR control for ACS patients when they concomitantly have hypertension.

## 1. Introduction

Heart rate (HR) is an easily measured and modifiable clinical parameter. Previous studies have demonstrated that HR was an independent risk factor for total and cardiovascular (CV) mortality in general population as well as in patients with CV disease [1–5]. In a post hoc analysis of the Platelet Glycoprotein IIb-IIIa in Unstable Angina: Receptor Suppression Using Integrilin Therapy (PURSUIT) trial, elevated HR was associated with 30-day death among patients with non-ST-segment elevation myocardial infarction (MI) [6]. In the morbidity-mortality evaluation of the I(f) inhibitor ivabradine in patients with coronary disease and left ventricular dysfunction (BEAUTIFUL) study, among patients with coronary artery disease (CAD) and left ventricular systolic dysfunction, every 5 beats per minute (bpm) increase of admission HR was associated with 8% increase in CV death, 16% increase in admission to hospital for heart failure, and 7% increase in admission to hospital for MI [7].

Elevated HR is common in patients with hypertension [8, 9]. According to the Hypertension and Ambulatory Recording Venetia Study (HARVEST), more than 15% young hypertensive subjects had a baseline HR ≥85 bpm and 27% had a HR ≥80 bpm [8]. Elevated HR has been found to be associated with the risk of mortality and adverse CV events in hypertensive patients regardless of with or without CAD [10, 11]. In the LIFE study, an increase of 10 bpm in HR was associated with a 25% increased risk of CV death and a 27% increased risk of all-cause mortality in hypertensive patients with left ventricular hypertrophy [11]. In the international verapamil SR-trandolapril study (INVEST), among patients with hypertension and CAD, an increase of 5 bpm in HR was associated with a 6% excess risk in adverse CV outcomes [12]. Although multiple studies have demonstrated the association of elevated HR with adverse CV outcomes in patients with hypertension and CAD, whether HR confers differential risk for adverse CV outcomes in CAD patients with versus without hypertension is not known. Therefore, the objective of this study was to assess the association of admission HR with major adverse CV events (MACEs) in patients with acute coronary syndrome (ACS) undergoing percutaneous coronary intervention (PCI) and to evaluate the value of admission HR modulated by hypertension as an independent predictor.

## 2. Materials and Methods

### 2.1. Population

This retrospective study consisted of all patients (*n* = 1770) with ACS undergoing PCI who were admitted to our CV center from June 2016 to November 2017. In terms of the purpose of this study, the exclusion criteria included nonsinus rhythm on the first electrocardiogram (ECG) after admission, taking *β*-blockers and other drugs that can significantly affect HR before admission, prior coronary artery bypass grafting, Killip class >2, connective tissue diseases, infectious diseases, thyroid dysfunction, and loss to follow-up. Finally, 1056 patients were included in the analysis. The present study involving human participants was in accordance with the Helsinki Declaration of Human Rights, and it was approved by the Medical Ethics Committee of Beijing Anzhen Hospital, Capital Medical University (2016034x). This retrospective study was considered minimal risk by the medical ethics committee; therefore, formal consent is not required. Written informed consent was obtained from all study participants after their admission.

### 2.2. Data Collection

Patient data on demographics, medical history, cardiovascular risk factors, laboratory assessments, and medical therapy at discharge were collected using a standard questionnaire. A standard questionnaire includes gender, age, BMI, smoking status, past medical history, pre-/posthospitalization medication history, and inpatient blood tests. Admission HR was defined as the HR recorded by the first available ECG after admission. ACS was diagnosed according to the American College of Cardiology/American Heart Association guidelines [13, 14]. Hypertension was defined as having at least two blood pressure recordings ≥140/90 mmHg and/or use of antihypertensive drugs. Diabetes was defined as symptoms of diabetes with a casual plasma glucose ≥11.1 mmol/l, fasting plasma glucose (FPG) ≥7.0 mmol/l, 2-hour plasma glucose ≥11.1 mmol/l from a 75 g oral glucose tolerance test, and/or use of antidiabetic drugs. Dyslipidemia was defined as a fasting serum total cholesterol >5.17 mmol/l, triglycerides >1.69 mmol/l, low-density lipoprotein cholesterol >3.36 mmol/l, high-density lipoprotein cholesterol <1.03 mmol/l, and/or use of lipid-lowering drugs.

### 2.3. Clinical Follow-Up and Outcomes

All patients were followed up at 1, 6, 12, 18, 24, 30, and 36 months after hospital discharge. The first participant was recruited in June 2016, and the follow-up ended in December 2019. Adverse CV events were obtained by trained personnel who never knew the baseline characteristics through telephone contact. The primary endpoint of this study was MACE, which was defined as the composite of all-cause death, nonfatal stroke, nonfatal MI, or unplanned repeat revascularization. Stroke was defined as ischemic cerebral infarction with lesions on computer tomography or magnetic resonance imaging and clinically corresponding neurological dysfunction. MI was defined as the levels of cardiac enzymes exceeding the upper limit with symptoms or ECG changes related to ischemia. Within 1 week after the index PCI, only Q-wave MI was defined as MI. Unplanned repeat revascularization was defined as any nonstaged revascularization after index PCI.

### 2.4. Statistical Analysis

The baseline characteristics of the study population were described according to the tertiles of admission HR. The first and third tertiles of admission HR were ≤66 bpm and ≥74 bpm, respectively (T1: HR ≤ 66 bpm, *n* = 369; T2: HR 67–73 bpm, *n* = 322; and T3: HR ≥ 74 bpm, *n* = 365). Continuous variables were reported as mean ± SD if consistent with a normal distribution, otherwise as median (0.25–0.75 percentiles). Categorical variables were reported as frequency and percentage. Comparisons among groups were performed using one-way ANOVA or Kruskal–Wallis H test for continuous variables and using the chi-square test for categorical variables. We used Kaplan–Meier curves to illustrate the cumulative incidence of MACE over time according to the tertiles of admission HR, and data were compared by using the log-rank test. The multivariate Cox proportional hazards regression model was used to assess the hazard ratio (HR) and 95% confidence interval (CI) for the relationship between admission HR and MACE. The interaction *P* value was examined between hypertension and admission HR (as a continuous variable using per 10 bpm increase or as a categorical variable using tertiles with the lowest tertile as the reference group). The model was built by stepwise variable selection to eliminate the multicollinearity between the variables. A total of 29 patient-specific baseline variables were initially screened for univariate association with clinical outcomes of interest at *P* < 0.15. Individual variables identified were then assessed in a forward stepwise manner with the use of a *P* value criterion of <0.05. Age, sex, admission HR, and hypertension were retained in the model regardless of *P* values. A two-side *P* value <0.05 was considered to be statistically significant. Statistical analyses were performed using the SPSS software (version 26, SPSS Inc., Chicago, Illinois).

## 3. Results

The baseline characteristics by the tertiles of admission HR are presented in Table 1. Patients with higher admission HR tertiles tended to be older, had higher rates of comorbidities, such as family history of CAD, hypertension, diabetes, dyslipidemia, and prior MI, and were more likely to have complex coronary lesions, such as three-vessel disease, proximal left anterior descending stenosis, bifurcation or trifurcation lesions, and heavy calcification lesions.

During the median follow-up period of 30 months, a total of 232 MACE events occurred. As demonstrated in Table 2, the patients with higher admission HR tertiles had higher incidence of MACE, as well as all-cause death, cardiovascular death, nonfatal MI, and unplanned repeat revascularization. The incidence of MACE was 16.5%, 19.3%, and 29.9% in T1, T2, and T3, respectively (*P* < 0.001).

After adjusting for other covariates in the multivariate Cox model, admission HR was an independent predictor of MACE, but hypertension was not. Compared with those in the lowest admission HR tertile, patients in the highest tertile had a 45.9% higher risk of MACE (adjusted HR: 1.459, 95% CI: 1.037–2.051, *P*=0.030) (Table 3). When stratifying the patients according to the presence or absence of hypertension, admission HR was significantly associated with a higher risk of MACE only in patients with hypertension (when T1 was taken as reference, the HR of T2 was 1.143 [95% CI: 0.700–1.864] and that of T3 was 2.062 [95% CI: 1.300–3.270]); however, in patients without hypertension, there was no significant correlation between admission HR and MACE (when T1 was taken as reference, the HR of T2 was 0.744 [0.406–1.364] and that of T3 was 0.614 [0.342–1.101]) (Table 3). There was a significant interaction for the risk of MACE between admission HR tertiles and hypertension (*P*=0.025 for interaction).

To further validate the interaction between admission HR and hypertension for the risk of MACE, we examined the associations of admission HR as a continuous variable with MACE in patients with versus without hypertensive in a fully adjusted multivariable model. In patients without hypertension, there was no relationship between admission HR and MACE (adjusted HR: 0.983, 95% CI: 0.957–1.009, *P*=0.194), whereas in hypertensive patients, each 10 bpm increase in admission HR was associated with a 42.3% increased risk of MACE (adjusted HR: 1.423, 95% CI: 1.089–1.765, *P*=0.002) (*P*=0.018 for interaction).

**Figure 1:**
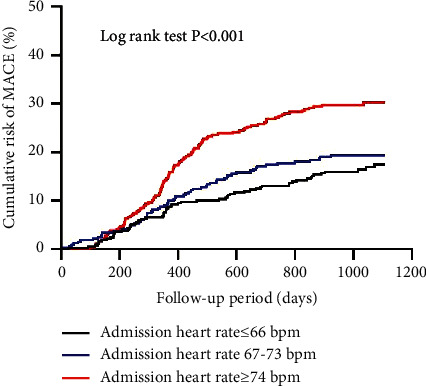
Kaplan–Meier curves of major adverse cardiovascular events stratified by admission heart rate tertiles.

**Figure 2:**
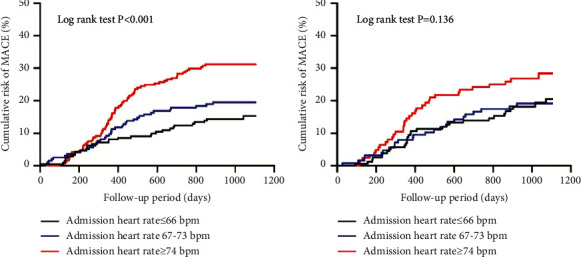
Kaplan–Meier curves of major adverse cardiovascular events stratified by admission heart rate tertiles among patients with (a) and without hypertension (b).

Figure 1 shows the cumulative incidence of MACE over time stratified according to admission HR tertiles in the overall population.  Figure 2 shows the cumulative incidence of MACE over time stratified according to admission HR tertiles among patients with (Figure 2(a)) versus without (Figure 2(b)) hypertension. In patients with hypertension, increasing tertiles of admission HR were associated with higher cumulative incidence of MACE over time (log rank *P* < 0.001); however, in those without hypertension, higher admission HR tertiles were not associated with higher cumulative incidence of MACE (log rank *P*=0.136).

## 4. Discussion

In this study, we examined the relationship between admission HR and MACE in ACS patients with versus without hypertension who were treated with PCI. We found that elevated admission HR, especially HR ≥74 bpm, was associated with a higher risk of MACE in patients with hypertension. Such relationship between HR and MACE no longer existed in patients without hypertension.

Multiple studies have indicated that HR is an independent predictor of CV morbidity and mortality. A meta-analysis showed that HR was significantly associated with the risk of CAD, stroke, and sudden death [15]; however, after studies involving patients with hypertension or diabetes were excluded, no association of HR with sudden death was found. The study of Diaz et al. showed that HR was a predictor of all-cause and CV mortality independent of other known risk factors such as hypertension, diabetes, and smoking in patients with suspected or proven CAD [5]. Timóteo et al. found that elevated admission HR was a predictor of mortality independent of left ventricular function in patients with ACS [16]. In the oral glycoprotein IIb/IIIa inhibition with Orofiban in Patients with Unstable Coronary Syndromes-Thrombolysis in Myocardial Infarction (OPUS-TIMI) 16 trial, higher initial and delayed HR was demonstrated to be highly predictive of higher short- and long-term mortality irrespective of time from onset of ACS [17]. Wang et al. found that in ACS patients who underwent PCI, HR > 76 bpm was associated with a higher risk of adverse CV events (cardiac death, nonfatal recurrent MI, ischemic-driven revascularization, or ischemic stroke) compared with HR 61–76 bpm during one-year follow-up, and an elevated HR ≥ 61 bpm was associated with increased risk of one-year adverse CV events [18]. Similarly, Noman et al. found that elevated admission HR in ST-elevation MI patients who underwent primary PCI was associated with increased risk of long-term mortality [19]. Similar to these previous reports, our study also confirmed that elevated HR was an independent predictor of adverse CV outcomes in patients with ACS undergoing PCI. Moreover, our study found that the independent association of admission HR with MACE occurred primarily in the individuals with hypertension but not in those without hypertension.

Several mechanisms may explain the association of elevated HR with MACE. In patients with CAD, elevated HR reduces left ventricular filling time and increases cardiac workload, resulting in an imbalance of oxygen supply and demand, and subsequently causing myocardial ischemia and angina [20]. In experimental studies, elevated HR has been shown to be associated with coronary atherosclerosis. Elevated HR can prolong the exposure of coronary endothelium to the systolic low and oscillatory shear stress and also lead to vascular oxidative stress, thereby promoting endothelial dysfunction and atherosclerosis [21–23]. On the contrary, lowering HR with ivabradine can reduce vascular oxidative stress and improve endothelial function, thereby preventing atherosclerosis [24, 25].

Hypertension has become a major public health issue worldwide, and its high incidence leads to the current pandemic of CV disease. Elevated HR has been shown to be independently associated with incident hypertension [26, 27] and an increased risk of heart failure in patients with hypertension [28]. Recent studies have shown that HR is partially controlled by the sympathetic nervous system and that elevated HR is a valid biomarker of sympathetic activation in essential hypertension [29, 30]. Previous studies have demonstrated that hypertensive patients with persistent HR ≥ 80 bpm have a higher risk of all-cause and CV death than those with HR < 80 bpm [31]. Elgendy et al. found that in CAD patients with hypertension and a history of heart failure, achieving systolic blood pressure of 120–140 mmHg and HR < 85 bpm was associated with a better prognosis [32]. These results may suggest combined effects of HR and hypertension on CV morbidity and mortality. Zhong et al. found that compared with normotensive patients with a HR < 80 bpm, hypertensive patients with a HR < 80 bpm and hypertensive patients with a HR ≥ 80 bpm were both at a higher risk of stroke and CAD [33]. Similarly, our study found that in patients with hypertension, compared with HR < 75 bpm, HR ≥ 75 bpm was associated with an increased risk of MACE; however, in patients without hypertension, no relationship between HR and MACE existed. These findings highlight the extra importance of HR control for ACS patients with hypertension.

Several limitations must be taken into account when interpreting the results of our study. First, the present study was a retrospective analysis derived from a prospective registry and only included 1056 patients which was a relatively small sample size. In addition, there were fewer female patients and a higher proportion of patients with unstable angina, which may lead to bias, but these two phenomena are consistent with the current medical situation in China. Second, the data of admission HR were obtained from the first ECG examination after admission. However, clinical factors such as admission time, onset-to-reperfusion time, and presence or absence of comorbidities could affect admission HR. Third, we excluded patients with prior coronary artery bypass grafting, Killip class >2, connective tissue diseases, infectious diseases, and thyroid dysfunction, patients with nonsinus rhythm on the first ECG after admission, and patients who were taking *β*-blockers, nondihydropyridine calcium channel blockers (CCBs), and other drugs significantly affecting HR before admission. Finally, the use of *β*-blockers and nondihydropyridine CCBs at discharge in some patients may affect the predictive value of admission HR for long-term outcomes; therefore, the results of our study may not be applicable to all patients with ACS. Large prospective studies are needed to explore the relationship between heart rate, time-varying heart rate, and MACE, and the potential impact of hypertension in a wider range of patients with ACS.

## 5. Conclusions

In patients with ACS undergoing PCI, the association of elevated admission HR with an increased risk of MACE was present in individuals with hypertension but not in those without hypertension. This finding suggests a potential benefit of heart rate control for ACS patients when they concomitantly have hypertension.

## Figures and Tables

**Table 1:** Baseline characteristics of the study population according to admission heart rate.

Variables	Admission heart rate tertiles			*P* value^*∗*^
	T1: ≤66 bpm (*n* = 369)	T2: 67–73 bpm (*n* = 322)	T3: ≥74 bpm (*n* = 365)	
Age (years)	58.7 ± 10.3	59.3 ± 9.9	61.1 ± 11.3	0.007
Female sex, *n* (%)	85 (23.0)	87 (27.0)	98 (26.8)	0.384
BMI (kg/m^2^)	25.2 (23.8–27.7)	25.2 (23.7–27.4)	25.1 (23.4–28.0)	0.837
Admission SBP (mm Hg)	129 ± 17	132 ± 16	131 ± 17	0.056
Admission DBP (mm Hg)	76 ± 10	77 ± 10	77 ± 12	0.049
Current smoking, *n* (%)	174 (47.2)	138 (42.9)	152 (41.6)	0.289
Family history of CAD, *n* (%)	107 (29.0)	95 (29.5)	123 (33.7)	0.324
Hypertension, *n* (%)	210 (56.9)	196 (60.9)	241 (66.0)	0.040
Diabetes, *n* (%)	146 (39.6))	143 (44.4)	188 (51.5)	0.005
Dyslipidemia, *n* (%)	286 (77.5)	256 (79.5)	314 (86.0)	0.009
CKD, *n* (%)	14 (3.8)	13 (4.0)	31 (8.5)	0.008
Prior MI, *n* (%)	41 (11.1)	54 (16.8)	65 (17.8)	0.025
Types of ACS, *n* (%)	—	—	—	—
UA	288 (78.0)	239 (74.2)	240 (65.8)	0.001
NSTEMI	45 (12.2)	38 (11.8)	61 (16.7)	0.105
STEMI	36 (9.8)	45 (14.0)	64 (17.5)	0.009
LVEF (%)	64 ± 6	63 ± 8	63 ± 8	0.102
Medications before admission, *n* (%)	—	—	—	—
Antiplatelet therapy	241 (65.3)	216 (67.1)	215 (58.9)	0.060
Statins	227 (61.5)	216 (67.1)	215 (58.9)	0.081
ACEIs/ARBs	101 (27.4)	67 (20.8)	86 (23.6)	0.127
DHP-CCBs	109 (29.5)	102 (31.7)	125 (34.2)	0.391
Insulin	39 (10.6)	50 (15.5)	69 (18.9)	0.006
Sulfonylurea	39 (10.6)	42 (13.0)	37 (10.1)	0.435
Metformin	35 (9.5)	30 (9.3)	64 (17.5)	0.001
* α*-Glucosidase inhibitors	28 (7.6)	36 (11.2)	41 (11.2)	0.173
Angiographic characteristics, *n* (%)	—	—	—	—
Left-main disease	0 (0.0)	2 (0.6)	0 (0.0)	0.102
Three-vessel disease	145 (39.3)	144 (44.7)	228 (62.5)	<0.001
Proximal LAD stenosis	147 (39.8)	156 (48.4)	218 (59.7)	<0.001
Bifurcation or trifurcation lesions	258 (69.9)	227 (70.5)	300 (82.2)	<0.001
Heavy calcification lesions	59 (16.0)	70 (21.7)	154 (41.4)	<0.001
Procedural results, *n* (%)	—	—	—	—
DES	305 (82.7)	273 (84.8)	315 (86.3)	0.390
BRS	45 (12.2)	13 (4)	7 (1.9)	<0.001
DCB	13 (3.5)	21 (6.5)	17 (4.7)	0.206
Complete revascularization	283 (76.7)	231 (71.7)	160 (43.8)	<0.001

*P* value^*∗*^: one-way ANOVA or Kruskal–Wallis H-test for continuous variables and chi-square test for categorical variables. BMI, body mass index; SBP, systolic blood pressure; DBP, diastolic blood pressure; CAD, coronary artery disease; CKD, chronic kidney disease; MI, myocardial infarction; ACS, acute coronary disease; UA, unstable angina; NSTEMI, non-ST-segment elevation myocardial infarction; STEMI, ST-segment elevation myocardial infarction; LVEF, left ventricular ejection fraction; ACEIs, angiotensin-converting enzyme inhibitors; ARB, angiotensin II receptor blocker; DHP-CCB, dihydropyridine-calcium channel blocker; LAD, left anterior descending.

**Table 2:** Chi-square tests for clinical outcomes during follow-up stratified by admission heart rate tertiles.

	Overall (*n* = 1056)	T1: ≥66 bpm (*n* = 369)	T2: 67–73 bpm (*n* = 322)	T3: ≥74 bpm (*n* = 365)	Log rank *P* value
MACEs, *n* (%)	232	61 (16.5)	62 (19.3)	109 (29.9)	<0.001
All-cause death, *n* (%)	33	6 (1.6)	6 (1.9)	21 (5.8)	0.002
Cardiovascular death, *n* (%)	24	4 (1.1)	6 (1.9)	14 (3.8)	0.037
Nonfatal stroke, *n* (%)	17	4 (1.1)	4 (1.2)	9 (2.5)	0.272
Nonfatal MI, *n* (%)	32	4 (1.1)	11 (3.4)	17 (4.7)	0.016
Unplanned repeat revascularization, *n* (%)	182	49 (13.3)	48 (14.9)	85 (23.3)	0.001

MACEs: all-cause death, nonfatal stroke, nonfatal MI, or unplanned repeat revascularization. MACEs, major adverse cardiovascular events; MI, myocardial infarction.

**Table 3:** Multivariate Cox proportional hazards analyses for MACEs according to the presence or absence of hypertension.

	HR (95% CI)	*P* value
*Overall population*		
Admission heart rate ≤66 bpm	Reference	—
Admission heart rate 67–73 bpm	1.024 (0.710–1.479)	0.897
Admission heart rate ≥74 bpm	1.459 (1.037–2.051)	0.030
Absence of hypertension	Reference	—
Presence of hypertension	0.967 (0.705–1.327)	0.835
*Absence of hypertension*		
Admission heart rate ≤66 bpm	Reference	—
Admission heart rate 67–73 bpm	0.744 (0.406–1.364)	0.339
Admission heart rate ≥74 bpm	0.614 (0.342–1.101)	0.102
*Presence of hypertension*		
Admission heart rate ≤66 bpm	Reference	—
Admission heart rate 67–73 bpm	1.143 (0.700–1.864)	0.594
Admission heart rate ≥74 bpm	2.062 (1.300–3.270)	0.002

MACE: all-cause death, nonfatal stroke, nonfatal MI, or unplanned repeat revascularization. MACEs, major adverse cardiovascular events; HR, hazard ratio; CI, confidence interval.

## Data Availability

The datasets generated during the current study are available from the corresponding author for reasonable request.
